# The role of type I interferon signaling in myeloid anti-tumor immunity

**DOI:** 10.3389/fimmu.2025.1547466

**Published:** 2025-03-03

**Authors:** Sofie Patrizia Meyer, Rebekka Bauer, Bernhard Brüne, Tobias Schmid

**Affiliations:** ^1^ Institute of Biochemistry I, Faculty of Medicine, Goethe University Frankfurt, Frankfurt, Germany; ^2^ Frankfurt Cancer Institute, Goethe University Frankfurt, Frankfurt, Germany; ^3^ Fraunhofer Institute for Translational Medicine and Pharmacology, Frankfurt, Germany; ^4^ German Cancer Consortium (DKTK), Partner Site Frankfurt, Frankfurt, Germany

**Keywords:** hypoxia, macrophage, phagocytosis, polarization, type I interferon, tumor microenvironment

## Abstract

Tumors often arise in chronically inflamed, and thus immunologically highly active niches. While immune cells are able to recognize and remove transformed cells, tumors eventually escape the control of the immune system by shaping their immediate microenvironment. In this context, macrophages are of major importance, as they initially exert anti-tumor functions before they adopt a tumor-associated phenotype that instead inhibits anti-tumor immune responses and even allows for sustaining a smoldering inflammatory, growth promoting tumor microenvironment (TME). Type I interferons (IFNs) are well established modulators of inflammatory reactions. While they have been shown to directly inhibit tumor growth, there is accumulating evidence that they also play an important role in altering immune cell functions within the TME. In the present review, we focus on the impact of type I IFNs on anti-tumor responses, driven by monocytes and macrophages. Specifically, we will provide an overview of tumor-intrinsic factors, which impinge on IFN-stimulated gene (ISG) expression, like the presence of nucleic acids, metabolites, or hypoxia. We will further summarize the current understanding of the consequences of altered IFN responses on macrophage phenotypes, i.e., differentiation, polarization, and functions. For the latter, we will focus on macrophage-mediated tumor cell killing and phagocytosis, as well as on how macrophages affect their environment by secreting cytokines and directly interacting with immune cells. Finally, we will discuss how type I IFN responses in macrophages might affect and should be considered for current and future tumor therapies.

## 1 Introduction

**Figure 1:**
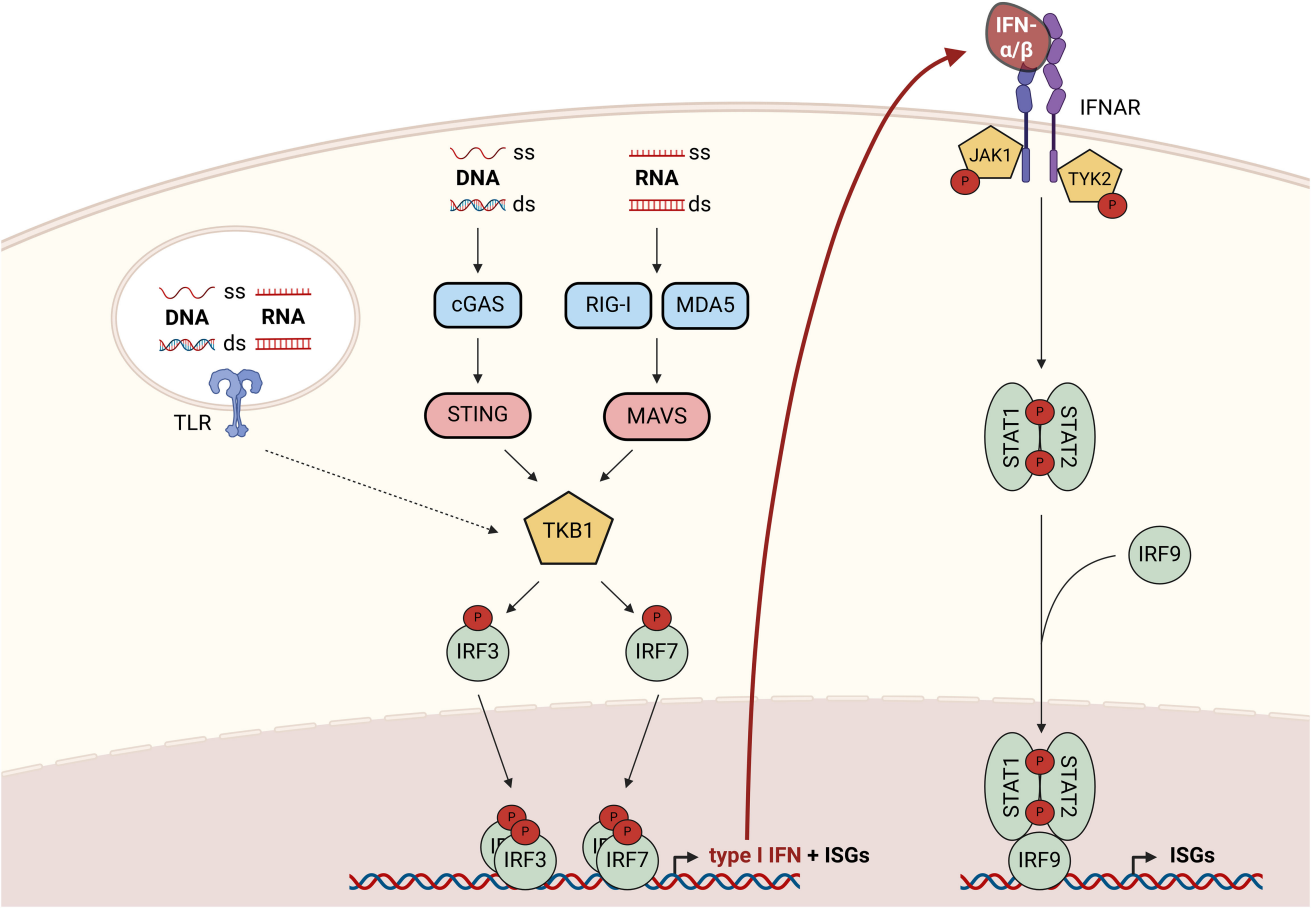
Overview of canonical signaling cascades regulating type I IFN production and responses to type I IFNs. Cytoplasmic nucleic acids activate TBK1 either via the cGAS-STING axis (ss or dsDNA) or via the RIG-I/MDA5-MAVS axis (ss or dsRNA). Upon activation, TBK1 phosphorylates and thereby activates the transcription factors IRF3 and 7 to promote the expression of type I IFNs and a subset of ISGs. Responses to extracellular nucleic acids are coordinated by endosomal TLRs (3, 7–9), which enable type I IFN production via TBK1-mediated IRF-activation. Upon secretion, type I IFNs bind to the IFNAR complex resulting in the activation of associated kinases JAK1 and TYK2, which phosphorylate and thereby activate STAT1 and 2. STAT1/2 homo- and heterodimers recruit IRF9 to form an active transcription factor complex enabling transcription of a broad panel of ISGs. cGAS, cyclic GMP-AMP synthase; ds, double-stranded; IFN, interferon; IFNAR, IFN-α/β receptor; IRF, interferon response factor; ISG, interferon-stimulated gene; JAK1, janus kinase 1; MDA5, melanoma differentiation-associated protein 5; MAVS, mitochondrial antiviral-signaling; RIG-I, retinoic acid-inducible gene 1; ss, single-stranded; STAT, signal transducer and activator of transcription; STING, stimulator of IFN genes; TBK1, TANK-binding kinase 1; TLR, toll-like receptor; TYK2, tyrosine kinase 2.

Type I interferons (IFNs), with the most prominent members IFN-α and IFN-β, are classically induced by viral infections to initiate anti-viral responses. Nevertheless, sterile inflammation, i.e., inflammatory response of pathogen-recognition receptors (PRRs) to non-pathogen-derived agonists such as intracellular contents from damaged, stressed, or dying cells, elicits type I IFN responses as well [for an overview readers are referred to (1)]. The production of type I IFNs is largely regulated by PRRs detecting foreign or self-nucleic acids in the cytoplasm or extracellularly (**Figure 1**). Canonical type I IFN induction in response to cytosolic double-stranded (ds), but also single-stranded (ss) DNA is mediated via the cyclic guanosine monophosphate–adenosine monophosphate (cGAMP) synthase (cGAS), which activates the stimulator of IFN genes (STING) (2, 3). ssRNA or dsRNA on the other hand are sensed by retinoic acid-inducible gene 1 (RIG-I)-like receptors (RLRs), such as RIG-I and melanoma differentiation-associated protein 5 (MDA5), that activate mitochondrial antiviral-signaling (MAVS) protein (4). Both STING and MAVS activate TANK-binding kinase 1 (TBK1), which subsequently phosphorylates interferon response factors (IRFs) 3 and 7. Upon homodimerization, the IRFs facilitate transcription of IFN-β and a subset of interferon-stimulated genes (ISGs) (5). Similarly, toll-like receptors (TLRs) 3, 7, 8, and 9 can induce expression of IFN-β and some ISGs via IRFs, when activated by extracellular nucleic acids within endo- and lysosomal compartments. While TLRs 2 and 4 rather respond to none nucleic acid-derived pathogen- or damage-associated molecular patterns (PAMPs/DAMPs), they can also elicit IFN responses [for an excellent, comprehensive review see (6)]. Of note, the tumor microenvironment (TME) contains various factors and conditions affecting type I IFN production, including adaptive mechanisms to limit excessive type I IFN levels (7). Upon secretion, type I IFNs activate the IFN-α/β receptor (IFNAR) complex comprised of IFNAR-1 and 2 subunits in an autocrine or paracrine manner. IFNAR is a janus kinase 1 (JAK1)/tyrosine kinase 2 (TYK2)-associated receptor, which phosphorylates signal transducer and activator of transcription (STAT) 1 and 2 upon ligand binding. Phosphorylated STAT1 and 2 form homo- or heterodimers and recruit IRF9 to enhance transcription of a broad panel of ISGs (8).

In this review, we will initially summarize the current understanding of environmental conditions and molecular mechanisms regulating type I IFN production by various cell types in the TME. Then, we will provide an overview of the impact of such elevated type I IFNs specifically on monocyte and macrophage phenotypes and functions within the TME.

## 2 Regulation of type I interferon production in tumors

**Figure 2:**
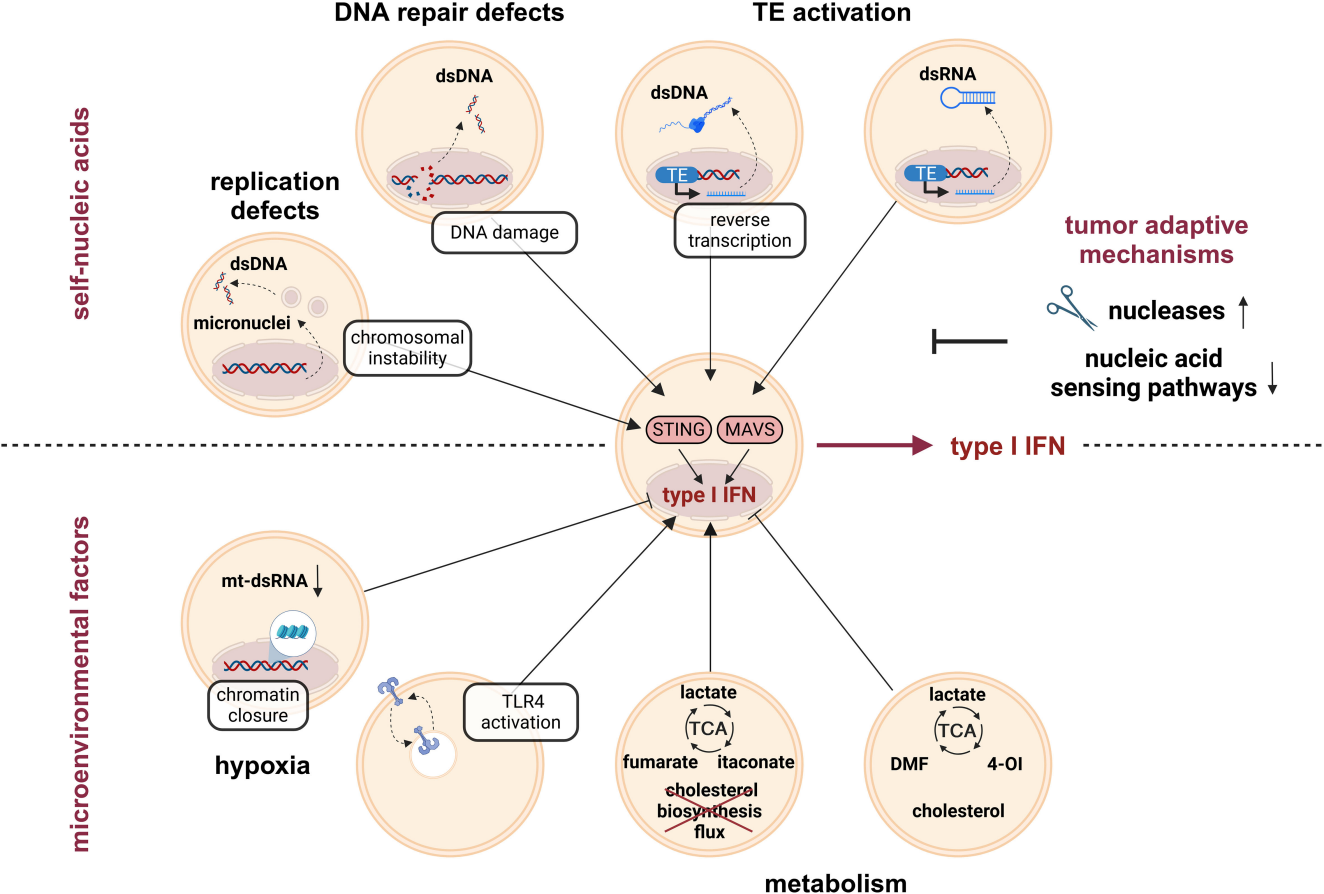
Type I IFN induction by tumor-intrinsic factors. Genome instability is a hallmark of tumors, which is responsible for accumulation of cytoplasmic self-nucleic acids. For example, replication defects and associated chromosomal instability, but also DNA damage due to defective DNA repair mechanisms trigger the release of dsDNA to the cytoplasm. Furthermore, activation of transposable elements (TE) contributes to the accumulation of immune-stimulatory cytoplasmic dsDNA intermediates (due to reverse transcription of the LINE1 retrotransposon). Cytoplasmic dsDNA activates STING and thereby enhances type I IFN production. In addition, TE activation contributes to the accumulation of cytoplasmic dsRNA intermediates. Cytoplasmic dsRNA activates MAVS and thereby contributes to elevated type I IFN production in tumors. In order to escape anti-tumor immune responses, tumors evolve mechanisms to prevent excessive type I IFN induction due to the release of self-nucleic acids, such as upregulation of nucleases or downregulation of nucleic acid-sensing pathways. Another type I IFN-regulating factor in the tumor microenvironment (TME) is reduced oxygen availability, i.e., hypoxia. While hypoxia decreased type I IFN production by reducing the amount of mt-dsRNAs and by diminishing chromatin accessibility in promotor regions with STAT1 and IRF3 binding motifs, respectively, other reports showed increased type I IFN production, mainly via TLR4 activation. Metabolic changes, among others induced by hypoxia, further regulate type I IFN production either positively (e.g., by fumarate, itaconate, or a disrupted cholesterol biosynthesis flux) or negatively (e.g., by the fumarate-derivate DMF, the itaconate-derivate 4-OI, or increased endoplasmic reticulum (ER) cholesterol levels). DMF, dimethyl fumarate; ds, double-stranded; IFN, interferon; IRF, interferon response factor; LINE1, long interspersed nuclear element 1; MAVS, mitochondrial antiviral-signaling; mt, mitochondrial; STAT, signal transducer and activator of transcription; STING, stimulator of IFN genes; TCA, tricarboxylic acid cycle; TLR, toll-like receptor; 4-OI, 4-octyl itaconate.

Tumors produce type I interferons due to typical features of the TME including the presence of self-nucleic acids or environmental factors such as hypoxic and metabolic changes (**Figure 2**).

### 2.1 Self-nucleic acids

In healthy cells, the subcellular localization of PRRs and the absence of self-nucleic acids in these compartments ensures that responses are usually restricted to foreign nucleic acids. However, in cancer cells various defects result in the presence of self-nucleic acids in the cytoplasm, causing the induction of type I IFN signaling, mainly through the cGAS/STING axis. In the following sections, we will summarize common tumor-associated alterations, which contribute to enhanced levels of self-nucleic acids in the cytoplasm (2.1.1) and explain how tumors successfully limit the associated production of type I IFNs (2.1.2) (**Figure 2**, upper part). As this review aims at primarily discussing the response of monocytes/macrophages to altered IFN levels in the TME independent of the exact source, we will only provide a broad overview of the factors contributing to elevated type I IFN production by both tumor and stromal cells within the TME. For more detailed pictures of individual aspects, we refer the readers to recent, comprehensive reviews [e.g., on genomic instability and inflammatory responses (9, 10) and on mitochondria-derived self-nucleic acids (11, 12)].

#### 2.1.1 Tumor-associated changes contributing to elevated self-nucleic acid levels

Genomic instability is a hallmark of cancer and refers to an overall increase in the frequency of mutations in a cell. In the following paragraph, we provide examples how genetic alterations and defects in genome maintenance result in the accumulation of mutations and release of dsDNA and thereby increase type I IFN production in the tumor cells, which consequently impacts macrophage-driven immune responses.

##### 2.1.1.1 DNA repair defects

Under physiological conditions, a coordinated interplay between cell cycle arrest, DNA repair mechanisms, damage tolerance, and initiation of cell death pathways protects the organism from the deleterious consequences of DNA damage. In cancer cells, however, these processes often fail to function properly, resulting in the leakage of ruptured DNA fragments into the cytoplasm and consequently, induction of type I IFNs. For example, a mutation in histone H3.3 (H3.3-G34r/V), which is found in approximately 16% of hemispheric pediatric and young adult high-grade gliomas, was shown to downregulate expression of DNA repair-related genes, thereby increasing the susceptibility to DNA damage, accumulation of cytosolic dsDNA, and the secretion of IFN-β (13). Along the same lines, deficiency of the mismatch repair protein MutL protein homolog 1 (MLH1), commonly observed in Lynch syndrome patients, who generally have a predisposition to develop a broad range of solid tumors, especially colorectal and endometrial cancer (14), leads to loss of regulation of exonuclease 1 (EXO1) activity during DNA repair. The consequences are uncontrolled DNA excision, generation of chromosomal abnormalities, release of nuclear DNA into the cytoplasm, and eventually induction of IFN-β through the cGAS/STING pathway (15, 16). Similarly, dysfunction of ataxia-telangiectasia mutated (ATM), a serine/threonine kinase critically involved in the response to and repair of DNA double-strand breaks, provokes the accumulation of damaged DNA in the cytosol, which results in constitutive STING-dependent type I IFN production, contributing to the inflammatory phenotype characteristic for the cancer-prone disease ataxia-telangiectasia (17). Interestingly, others proposed non-canonical STING activation by ATM after DNA damage in human keratinocytes. Specifically, the authors postulate that DNA damage-induced, ATM-dependent formation of a signaling complex, consisting of IFI16, p53, TRAF6, and STING, induces non-canonical NF-κB-mediated IFN-β production (18). A study on non-small cell lung cancer (NSCLC) further demonstrated that defective DNA repair mechanisms do not always correlate positively with the production of type I IFNs. In this system, the proto-oncogene EMSY was stabilized in kelch like ECH associated protein 1 (KEAP1) mutant tumors, which in turn inhibited homologous recombination repair (HRR), causing high tumor mutational burden that would normally be expected to foster type I IFN induction. However, EMSY appeared to repress the induction of IFN-β and ISGs, thus providing an explanation for immune evasion in KEAP mutant NSCLC despite high levels of DNA damage (19). Taken together, DNA repair defects commonly result in the accumulation of dsDNA, which again stimulates type I IFN production.

##### 2.1.1.2 Activation of transposable elements

Transposable elements (TEs), such as DNA transposons and retrotransposons, are mobile DNA sequences, which make up around 45% of mammalian genomes (12). Since integration of TEs in new gene loci bears the risk for gene disruptions and mutagenic effects, TE expression is normally silenced in mammalian adult tissues, especially by epigenetic mechanisms such as DNA methylation. However, in cancer cells TEs commonly become active. Beside the formation of deleterious mutations and chromosomal rearrangements, TE activation and relocation can also induce the accumulation of self-nucleic acids and as a consequence the production of type I IFNs. Specifically, the absence of the tumor suppressor p53 in combination with DNA hypomethylation was shown to cause massive transcription of the major classes of usually silent short, interspersed nuclear elements (SINEs) B1 and B2, as well as of numerous non-coding RNAs (ncRNAs). The latter appeared to form dsRNA structures with the respective SINEs, resulting in enhanced MAVS-dependent type I IFN production (20). In addition to SINEs, also active long interspersed nuclear element 1 (LINE1) was shown to stimulate *IFNB* mRNA expression (21). Mechanistically, in aged mice increased LINE1 activation and reverse transcription resulted in accumulation of cytoplasmic LINE1 cDNA, which triggered type I IFN responses (22). For further details on TEs and IFNs, we refer interested readers to a recent, comprehensive review (23).

##### 2.1.1.3 DNA replication defects

Chromosomal instability (CIN), characterized by the gain or loss of entire chromosomes, arises from errors in DNA replication or chromosome segregation during cell division, and causes formation of micronuclei. These are prone to rupture and, thus, spill their DNA contents into the cytoplasm [for a comprehensive overview see (24)]. As demonstrated in several acute myeloid leukemia (AML) cell lines treated with a CIN-inducing monopolar spindle 1 (MPS1) kinase inhibitor, micronuclei generation, activation of cGAS/STING signaling, and induction of IFN-β are the consequences of chromosome mis-segregation (25). Of note, cGAS even specifically localizes to micronuclei following DNA damage or nuclear envelope rupture (26). CIN also arises, if reduced levels of the DnaJ heat shock protein family member A2 (DNAJA2) are expressed. Since DNAJA2 regulates chaperone-mediated autophagy of centriolar satellite proteins, DNAJA2 deficiency results in mitotic defects, CIN, and consequently type I IFN induction (27). Yet, there is emerging evidence that some tumors have established mechanisms to prevent CIN-induced inflammatory responses to evade the immune system [nicely reviewed in (28)].

#### 2.1.2 Tumor strategies to limit induction of type I IFNs by self-nucleic acids

While the immune system initially keeps degenerated cells in check, tumors eventually acquire properties enabling them to escape adverse immune responses, a process termed immune evasion (29). In line, IFN responses are generally considered to exert anti-tumor functions, yet tumors evolve to either reduce IFNs or become insensitive to elevated IFN levels (30).

##### 2.1.2.1 Nuclease-mediated degradation of self-nucleic acids

One mechanism to evade immune surveillance owing to genomic instability or TEs is the degradation of self-nucleic acids by nucleases. A prominent candidate in the class of nucleases is the 3′-5′ DNA exonuclease three prime repair exonuclease 1 (TREX1) that degrades cytosolic DNA from different sources. In the context of inflammatory autoimmune diseases, such as systemic lupus erythematosus (SLE) or Aicardi-Goutières syndrome (AGS), it is well established that TREX1 deficiency results in aberrant cGAS/STING-mediated type I IFN production, underlining the important IFN-regulatory function of TREX1 (31, 32). Thus, it is not surprising that tumor cells induce TREX1 upon CIN in a cGAS/STING-dependent manner as an adaptive feedback mechanism to promote removal of cytosolic DNA, and thus, allow for immune evasion. TREX1-deficient cancer cells showed elevated IFN-β production and consequently reduced tumor growth (33). Indeed, TREX1 appears to be upregulated in different tumor entities, including cervical, breast, and hepatocellular cancer (34, 35), making TREX1 an attractive target in tumor therapy, whose inactivation is thought to increase cancer cell immunogenicity (36, 37). Other nucleases, such as DNAse II or adenosine deaminase acting on RNA 1 (ADAR1), are known to limit type I IFN production by degrading cytosolic IFN-inducing nucleic acids as well (38, 39). Along these lines, activity of the helicase DEAD-box helicase 3 X-linked (DDX3X) was observed to be important in preventing the accumulation of cytoplasmic dsRNA and subsequent IFN induction via MDA5 (40).

##### 2.1.2.2 Downregulation of nucleic acid-sensing pathways in cancer

Alternatively, tumors can adapt by downregulating nucleic acid-sensing pathways to avoid tumor-suppressive IFN production. In the context of altered sensing systems, cGAS/STING signaling was shown to be defective in numerous cancer cells. For example, many colorectal and melanoma cancer cell lines responded to transfection of dsDNA with only weak or non-abundant IFN-β secretion, while dsRNA-triggered RIG1 pathway appeared to be intact in most cell lines. As a reason for the unresponsiveness, the authors identified cGAS silencing through promotor hypermethylation (41–43). Pointing in a similar direction, treatment of STING-defective human melanoma cell lines with the DNA methyltransferase inhibitor 5-aza-2’-deoxycytidine (5AZADC) reinstated functional STING signaling and IFN-β secretion after 2,3-cGAMP treatment (44). In addition to epigenetic mechanisms, JAK2/STAT3 signaling was recently shown to be responsible for STING deactivation and reduced type I IFN expression in the prostate cancer cell line DU145, although the exact molecular mechanisms remain elusive (45).

In summary, several tumor-intrinsic mechanisms cause the release of self-nucleic acids, thereby initiating the production of type I IFNs. To avoid overt anti-tumor responses, tumor cells commonly either degrade self-nucleic acids or deactivate crucial sensing systems, such as cGAS/STING signaling, thereby minimizing the production of type I IFNs and associated anti-tumor immune responses.

### 2.2 Tumor microenvironmental factors

In addition to altered detection of self-nucleic acids, tumor microenvironmental cues impinge on type I interferon production in tumors as well, including reduced oxygen availability, i.e., hypoxia, (2.2.1) and metabolic adaptations (2.2.2) (**Figure 2**, lower part).

#### 2.2.1 Hypoxia

Solid tumors are commonly associated with rapid growth and an inadequate supply of nutrients and oxygen due to insufficient vascularization. Consequently, most solid tumors contain areas exposed to chronic intermittent hypoxia, which shape the tumor microenvironment. Although it is now widely accepted that oxygen deprivation influences the production of type I IFNs as well as the response to these, the exact regulation appears to be highly dependent on the cellular and environmental context. For instance, Miar and colleagues not only observed reduced ISG expression levels under hypoxia, but also lower poly(I:C)-induced IFN production under hypoxic incubation in human breast cancer cells. Hypoxic desensitization was attributed to reduced chromatin accessibility in promotor regions with STAT1 and IRF3 binding motifs, and thus, repressed transcription of various players of the type I IFN pathway (46). Subsequently, reduced mitochondrial (mt) DNA transcription and consequently reduced levels of immune-stimulatory mt-dsRNA under hypoxia were proposed as an additional mechanism of how tumor cells reduce type I IFN signaling in response to hypoxia. Interestingly, mice housed for 24 h under low oxygen conditions, causing hypoxemia, displayed lower blood, i.e., systemic, IFN-α levels after lipopolysaccharide (LPS) challenge than normoxic housed mice (47). Similarly, hypoxia reduced IFN-α production and expression of selected ISGs in response to stimulation with TLR4-activating high mobility group box 1 (HMGB1) in primary monocytes and macrophages, while oxygen deprivation without an inflammatory stimulus appeared to provoke the opposite (48). In line with the latter observation, low oxygen levels have also been shown to increase type I IFN production. Such apparently contradictory findings regarding the impact of hypoxia on type I IFN production can be attributed largely to differences in study design (e.g., duration and degree of hypoxia, additional inflammatory stimuli, cell type, metabolic state of the cells, or *in vivo* conditions). For instance, cerebral ischemia induced expression of RIG-1 and IFN-α in rats, which could be reproduced *in vitro* by depriving astrocytes of both oxygen and glucose (49). Upregulated RIG-1 and type I IFNs were also found in rat kidney cells, in mouse kidneys, and skeletal muscle cells under hypoxia (50, 51). We recently observed that hypoxia induced IFN-β and consequently broad ISG expression in monocytic cells, via enhanced TLR4 signaling, which resulted from altered membrane dynamics and intracellular trafficking processes of PRRs due to changes in cholesterol homeostasis (52). Along the same lines, McDonough and colleagues observed TLR4-dependent, IFNAR-mediated upregulation of ISGs in microglia *in vitro* in response to ischemia/reperfusion. Interestingly though, ISG induction in *in vivo* ischemia/reperfusion experiments appeared to be independent of TLR4 (53). In contrast to the effects observed in myeloid cells, hypoxia decreased IFN-β response upon infection with the amoeba *Acanthamoeba* in human corneal epithelial cells by diminishing TLR4 signaling (54).

Considering the tight association of hypoxic environments with tumors, but also with inflammatory processes in general, there is need for further studies to comprehensively characterize the impact of hypoxia on type I IFN production and signaling in different cell types *in vitro* and under pathophysiological situations *in vivo*, also with respect to the role of TLR4. For a more comprehensive overview of the impact of hypoxia on IFNs, we refer interested readers to a recent, comprehensive review (55).

#### 2.2.2 Metabolism

As already proposed 100 years ago, cancer cells undergo metabolic remodeling, also known as the Warburg effect (56). Specifically, even in the presence of oxygen, cancer cells preferentially utilize glycolysis with concomitant production of lactate over oxidative phosphorylation for ATP generation. This is driven largely by the extensive uptake of metabolites, such as glucose. In the meantime it was shown that not only glucose metabolism, but also most other metabolic pathways are altered in tumors [for excellent reviews readers are referred to (57–59)]. Not surprisingly, metabolic changes are also tightly intertwined with immune responses, a research field coined “immunometabolism” in 2011 (60). It appears rational to speculate that the adapted metabolic phenotype in tumors and its microenvironment might influence the production of type I IFNs as well.

In line, lactate was shown to inhibit RLR-mediated type I IFN induction *in vitro* and *in vivo* (61). Similarly, lactate reduced IFN-α induction in response to TLR ligands in plasmacytoid dendritic cells (pDCs) (62). The contradictory finding that lactate increased mtDNA-triggered cGAS/STING-dependent type I IFN production in monocytic THP-1 cells was attributed to enhanced lactylation of cGAS, which prevented its ubiquitination and degradation, and consequently lead to stronger type I IFN responses (63). Thus, the effect of lactate on type I IFN induction appears to largely depend on the involved PRR. Considering that type I IFN induction in the TME is initiated predominantly via the cGAS/STING signaling pathway, it appears likely that lactate accumulation increases rather than decreases type I IFN levels in the TME.

In addition to lactate, TCA cycle-derived metabolites, especially itaconate and fumarate, were described to influence type I IFN production. After LPS stimulation, macrophages produce high amounts of itaconate from the TCA cycle intermediate aconitate via aconitate decarboxylase 1 (ACOD1), which is encoded by immune-responsive gene 1 (*Irg1*) (64). In fact, itaconate was one of the most upregulated metabolites in peritoneal tumors of mice and increased *Irg1* mRNA expression was found in human monocytes derived from ovarian carcinoma ascites (65). Interestingly, the classical LPS-induced ISG signature was diminished in *Irg1* ^-/-^ murine bone marrow-derived macrophages (BMDMs) and could be restored again by itaconate supplementation. Moreover, itaconate pre-treatment further enhanced IFN-β levels upon LPS stimulation (66). Thus, elevated itaconate levels in tumors might contribute to type I IFN production in myeloid cells within the TME. Of note, itaconate derivatives, such as 4-octyl itaconate (4-OI), show a functional overlap with itaconate in many biological contexts, but display opposite effects in their immunomodulatory function (66). Similar to itaconate, fumarate was described to induce IFN-β and ISGs in macrophages, supposedly via a release of mtRNA and subsequent activation of TLR7, RIG-1, and MDA5 (67), while the fumarate derivative dimethyl fumarate (DMF), alike to 4-OI, reduced IFN-β levels in an inflammatory context (68, 69). Along similar lines, fumarate-associated mitochondrial remodeling was suggested to induce type I IFNs via the activation of cGAS/STING signaling by mtDNA (70). Considering that fumarate was proposed to play an important role in the development of cancer (71), it will be interesting to uncover more details on how fumarate, but also other TCA cycle-derived metabolites, might affect type I IFN signaling and consequently the TME.

Furthermore, lipid metabolism, particularly cholesterol metabolism, is highly connected to inflammatory responses in general and specifically to type I IFN signaling (52). For example, limiting the flux through the cholesterol biosynthesis cascade induced the expression of IFN-β and ISGs in BMDMs (72). Along these lines, perturbation of the flux due to supplementation of the cholesterol precursor 7-dehydrocholesterol or deficiency of its synthesizing enzyme 7-dehydrocholesterol reductase (DHCR7) augmented IFN-β production upon infection (72). Similarly, we previously observed that hypoxia-altered cholesterol homeostasis induced expression of IFN-β and ISGs in an AML cell line (73). A recent study provided further evidence for the tight interconnection between cholesterol and IFNs. Specifically, they showed that STING contains two cholesterol-binding motifs (so called cholesterol recognition/interaction amino acid consensus (CRAC/CARC) motifs), which retain STING in the ER. Surprisingly, cGAMP stimulation reduced ER cholesterol levels, thereby allowing STING to traffic from ER to Golgi, where it contributed to type I IFN production in monocytic cells (74). Of note, several TLRs also contain CRAC motifs (75), hinting towards a general cholesterol-dependent mode of regulating PRR responses. Considering that increased cholesterol biosynthesis is a hallmark of many tumors (76), alterations in cholesterol metabolism are likely to have a significant impact on the amount of secreted type I IFN in the TME.

Taken together, there is ample evidence that numerous metabolic alterations in the TME impinge on type I IFN signaling. For a more detailed overview of the immunometabolic aspects of type I IFN production, readers are referred to a recent comprehensive review (77).

## 3 Effect of type I interferons on macrophages in the tumor microenvironment

**Figure 3:**
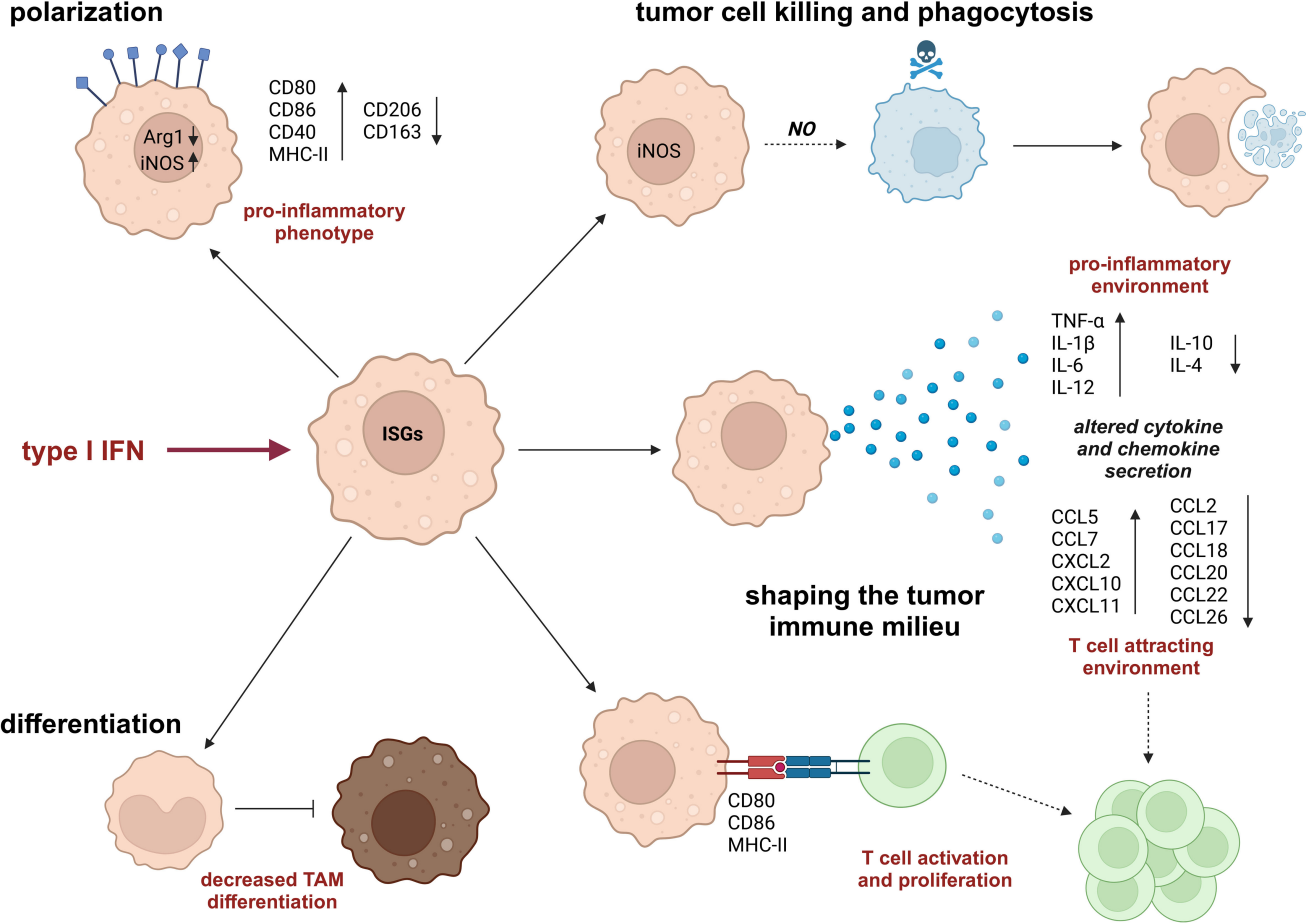
Impact of type I IFN on monocyte/macrophage phenotypes and functions. Type I IFNs modify monocyte and macrophage phenotypes by altering their differentiation pattern, e.g., reducing differentiation of monocytes to tumor-associated macrophages (TAMs) and increasing pro-inflammatory polarization characteristics. In addition, type I IFNs affect macrophage functions by enhancing their tumoricidal and phagocytic capacities and shaping a tumor-suppressive tumor microenvironment (TME), which is characterized by increased levels of pro-inflammatory cytokines and T cell-attracting chemokines as well as by a direct, activating impact on T cells. Arg1, arginase 1; CCL, C-C motif chemokine-ligand; CD, cluster of differentiation; CXCL, C-X-C motif chemokine ligand; iNOS, inducible nitric oxide synthase; IL, interleukin; MHC, major histocompatibility complex; NO, nitric oxide; TNF, tumor necrosis factor.

Myeloid cells, especially monocytes and macrophages, are an important part of the immune infiltrate in tumors. While in certain tumor types (e.g., glioblastoma), macrophages are the main constituents in this context, they play a dual role in tumorigenesis, i.e., they can contribute to anti- but also pro-tumor functions depending on the specific TME. Even though TME-associated conditions are well-known to alter the production of type I IFNs, how exactly such altered type I IFN levels affect macrophages in the TME remains only partly characterized. In the following section, we will therefore provide an overview of the current understanding of the impact of type I IFN responses on macrophage differentiation (3.1), polarization (3.2), and function (3.3) in the tumor context (**Figure 3**). Importantly, most of the factors, which induce type I IFN production in the TME, can also directly affect macrophage functions (e.g., hypoxia). Yet, the potential contribution of type I IFNs to these supposedly direct effects remains only poorly characterized.

### 3.1 Macrophage differentiation

Based on their origin, there are two types of macrophages: Monocyte-derived macrophages are recruited from the periphery and enter the site of acute inflammation, while tissue-resident macrophages initially are derived from embryonic precursors (like yolk sac or fetal liver) and differ between tissue types, for example microglia are found in the brain, Kupffer cells in the liver, or Langerhans cells in the skin [as comprehensively reviewed in (78)]. Nevertheless, some tissue-resident macrophages, like bone-resorbing osteoclasts, also have a hematopoietic origin (79). Upon infection or injury, circulating monocytes are recruited to the site of inflammation and quickly differentiate into macrophages or DCs, depending on the inflammatory signals. In line, it is well-established that *in vitro* monocytes are differentiated into macrophages with either macrophage colony-stimulating factor (M-CSF) or granulocyte–macrophage colony-stimulating factor (GM-CSF), while stimulation with GM-CSF and IL-4 induces differentiation into DCs (80). Replacement of IL-4 with type I IFN results in DC differentiation of a plasmacytoid phenotype instead (81). In a transplanted murine tumor model, enhanced type I IFN signaling in response to TLR3 stimulation with poly(I:C) reduced the number of tumor-associated macrophages (TAMs) in these tumors as well. This effect was attributable to attenuated monocyte-to-macrophage differentiation, which could be rescued by the inhibition of IFNAR (82, 83). Similarly, an increased number of TAMs was observed in fibrosarcoma tissue from IFNAR knock-out (KO) mice compared to WT mice, indicating that type I IFN signaling is involved in the suppression of TAM generation (84). Alternatively, it can be speculated that type I IFN signaling simply shifts monocyte differentiation from macrophages to DCs in the tumor context. Furthermore, ubiquitin-specific peptidase 18 (USP18), a negative regulator of type I IFN signaling, appeared to be critical for differentiation of tumor-supportive TAMs in B16F10 melanomas (85). In line, intratumoral injection of the STING agonist cGAMP enhanced the recruitment of anti-tumorigenic macrophages to the tumor site (86). Thus, type I IFNs appear to suppress the differentiation into tumor-supportive TAMs.

Besides, type I IFNs can also affect tissue-resident macrophages in the TME. For example, it is known that type I IFNs inhibit osteoclastogenesis (87). Accordingly, increased IFN-β expression in prostate cancer cells was found to downregulate osteoclast activation genes like receptor activator of NF-κB (RANK) and matrix metalloproteinase-9 (MMP9) in RAW264.7 precursor cells co-cultured with tumor cells (88). Wang and colleagues further assumed an inhibition of osteoclastogenesis after treatment of tumor-bearing mice with STING agonists due to a reduced number of osteoclasts in the bone TME, supported by similar observations *in vitro* with RAW264.7 macrophages or BMDMs (89). Moreover, intracranial administration of IFN-β gene therapy increased numbers of activated microglia in the tumor-surrounding tissue based on the expression of Iba1 and the amoeboid morphology of microglia (90). The continuous secretion of IFN-β by engineered mesenchymal stem cells in brain tumors derived from co-implanted tumor cells also resulted in slightly decreased macrophage numbers in tumors and increased frequency of microglia, especially activated microglia expressing major histocompatibility complex class II (MHC-II) (91).

In conclusion, type I IFNs not only attenuate the differentiation of monocytes into macrophages, but also alter the activation of both (which will be discussed below). Thereby, type I IFNs affect monocyte-derived, infiltrating macrophages as well as replenishing tissue-resident macrophages, both of which are present and important in the TME.

### 3.2 Macrophage polarization

Mature macrophages exhibit a huge heterogeneity and plasticity with numerous subtypes due to their ability to polarize into different states with different functional properties. Traditionally, macrophages were classified into classically activated (M1-like) macrophages, which initiate and sustain inflammation and are involved in tumor cell killing and tissue damage. Alternatively activated (M2-like) macrophages on the other hand promote resolution of inflammation and tissue repair, but also contribute to chronic smoldering inflammatory conditions, as commonly observed in tumors [for a detailed overview see (92)]. Owing to their enormous plasticity and the wealth of observed phenotypes, the original M1/M2 nomenclature of macrophages has been adapted to directly associate respective phenotypes with distinct stimuli (93). Moreover, *in vivo* macrophages commonly appear to take on intermediate phenotypes expressing markers of various polarization states (93). Today, single-cell resolution technologies provide valuable tools to characterize the broad spectrum of TAMs. Of note, macrophage polarization often occurs already during the differentiation process and is indistinguishable from it. However, fully differentiated and polarized macrophages can also undergo re-polarization from one phenotype to the other (94).

In contrast to the impact of IFN-γ, the effect of type I IFNs on macrophage polarization remains only partly understood. Generally, type I IFNs are believed to contribute to M1-like pro-inflammatory states [reviewed in (95)]. Accordingly, the classical indicators of type I IFN signaling, interferon induced protein with tetratricopeptide repeats 1 (IFIT1), 2, and 3, were massively upregulated upon M1-like activation of differentiated THP-1 monocytic cells and M-CSF-differentiated macrophages derived from primary human monocytes (96). Moreover, IFN-β stimulation was shown to inhibit IL-4-induced M2-polarization by reducing the availability of α-ketoglutarate (97). In contrast though, BMDMs displayed a M2-like phenotype upon stimulation with IFN-β-containing supernatants from B cells isolated from mice infected with *Mycobacterium tuberculosis* (98). While the underlying differences await further characterization, selective activation of IRFs was suggested to contribute to differential macrophage polarization (99, 100). Specifically, while IRF2, 3, and 4 appear to be associated with immunosuppressive polarization, IRF1, 5, and 8 are rather involved in classical macrophage activation. To what extent the involvement of IRFs in macrophage polarization indeed reflects effects of type I IFNs remains to be elucidated.

Importantly, within the TME macrophages commonly take on an alternatively activated, immunosuppressive, tumor-supporting phenotype, often induced by signals originating directly from the tumor cells [for more details see (95)]. In line with the impact of type I IFNs on macrophage polarization in inflammatory settings, stimulation of macrophages (and monocytes) with type I IFNs within the TME was also proposed to shift them towards an inflammatory phenotype, associated with anti-tumor activities of the macrophages themselves, but also of other immune cells. Accordingly, induced pluripotent stem cell (iPSC)-derived macrophages overexpressing IFN-α or -β exhibited reduced expression of the M2-like macrophage and TAM marker CD206 (101). Moreover, treatment of hepatocellular carcinoma (HCC)-bearing mice with IFN-α shifted TAM phenotypes from an M2-like to an inflammatory polarization, characterized by decreased expression of CD206 and arginase 1 (Arg1) and increased expression of inducible nitric oxide synthase (iNOS) (102). Furthermore, the global knockout of IFNAR in glioblastoma-bearing mice, which predominantly altered the transcriptome of myeloid cells in the TME, increased RNA expression of Arg1 and CD206 (103). Depletion of the negative regulator of type I IFN signaling USP18 in BMDMs or supplementation of IFN-β also decreased CD206 expression and scRNA-seq analyses underscored an anti-tumor polarization of macrophages due to USP18 deletion (85). Macrophages engineered to produce IFN-α directly in the TME of liver metastatic lesions further showed an upregulated expression of genes related to antigen presentation like CD40 and MHC-II contributing to the eradication of the metastases (104). Furthermore, low doses of both IFN-α or IFN-β upregulated CD169 expression in macrophages (105). In line, Affandi and co-workers reported a dose-dependent induction of CD169 expression by IFN-α in CD14^+^ monocytes, as well as higher human leukocyte antigen (HLA)-DR expression in CD169^+^ monocytes among peripheral blood mononuclear cells (PBMCs) of cancer patients (106). While CD169 is not a typical polarization marker, CD169^+^ macrophages were shown to produce pro-inflammatory chemokines and contribute to anti-tumor responses (107).

In addition to type I IFNs themselves, various stimuli of type I IFN signaling were demonstrated to induce a shift towards classical macrophage activation in the TME. For example, stimulation of M2-pre-programmed BMDMs with the TLR3 agonist poly(I:C) was shown to downregulate CD206 and slightly upregulate CD80, CD86, and CD40, i.e., markers of an inflammatory macrophage phenotype. This switch was prevented either upon blockade of IFN-α or -β, or in BMDMs lacking IFNAR and was reproducible with TAMs isolated directly from colon adenocarcinomas from poly(I:C)-treated mice (108). As discussed above, the cGAS/STING pathway is one of the major regulators of type I IFN production and, thus, was also interrogated regarding its impact on macrophage polarization in the TME. Indeed, STING signaling was reduced in various tumor entities, including breast and lung carcinoma (109, 110). Activation of STING in macrophages resulted in enhanced type I IFN production, yielding decreased expression of M2-like markers (e.g., IL-10, Arg1) and increased expression of pro-inflammatory markers (e.g., IL-1β, TNF-α), consequently unleashing the anti-tumor properties of the macrophages (111). Ao and colleagues confirmed that pro-inflammatory polarization of macrophages in the TME can be achieved by a type I IFN response. Specifically, stimulation with a STING agonist allowed for a phenotype switch towards an anti-tumor macrophage phenotype, which, in combination with the concomitant enhanced antigen presentation by DCs and infiltration of cytotoxic T lymphocytes (CTLs), inhibited outgrowth of residual tumor (112). Likewise, combined inhibition of mitogen-activated protein kinase kinase (MEK) and autophagy triggered STING-mediated type I IFN production in pancreatic ductal adenocarcinoma cells, resulting in IFNAR-dependent re-polarization of TAMs towards a classically activated phenotype (113). In line, nanoparticle-mediated delivery of the STING agonist cGAMP into murine breast tumors decreased the expression of markers associated with alternatively activated macrophages (Ym1, Fizz1, Arg1, CD206) in isolated CD11b^+^ macrophages (114).

Despite the fact that cancer patients are frequently (co-)treated with type I IFNs, little information is available on the direct impact on tumor infiltrating immune cells. Interestingly, Kakizaki and co-workers found an increase in CD163^+^ macrophages without changes in CD206^+^ macrophage abundance in lesional skin of melanoma patients after peritumoral IFN-β administration. Functionally, IFN-β shifted the macrophage secretome from a Th2 (pro-tumor) to a Th1 (anti-tumor) response (115). Further conclusions about the polarization state of monocytes after type I IFN treatment of tumor patients can be drawn largely based on available blood analyses. In fact, numerous studies hint towards an anti-tumor re-polarization of monocytes and macrophages upon treatment of tumor patients with type I IFNs. For instance, while isolated blood monocytes from patients with small cell carcinoma of the bronchus displayed reduced HLA-DR expression compared to healthy donors, a five-day treatment with IFN-α increased its expression (116). Similarly, increased HLA-DR, HLA-DQ, and β2 microglobulin expression was observed in monocytes of IFN-β-treated patients with metastatic renal carcinoma (117). Furthermore, increased expression of CD86, CD40, and further costimulatory molecules was found in blood monocytes from stage IV melanoma patients after treatment with IFN-α (118).

While there is ample information supporting an anti-tumor macrophage phenotype upon type I IFN treatment of tumor patients, contrary findings have also been published. For example, extracellular vesicles (EVs) from triple negative breast cancer cell lines induced STING-dependent type I IFN responses in monocytes, and in parallel lead to the differentiation of monocytes into M2-like TAMs, based on the expression of relevant receptors such as CD206, CD163, and proto-oncogene tyrosine-protein kinase MER (MerTK). Importantly though, the authors did not establish an unambiguous causal link between the two observations (119). In line with these findings, Tong et al. not only demonstrated attenuated monocyte-to-macrophage differentiation after IFN-β- and poly(I:C)-treatment of Lewis lung carcinoma (LLC)-bearing mice, but also observed a marked induction of the immunosuppressive marker Arg1 in TAMs (82). The anti-inflammatory skewing of TAMs by poly(I:C) and IFN-β was further characterized to depend on IFNAR, IL-4, IL-6, and the Src homolog domain-containing phosphatase 2 (SHP2)/extracellular signal-regulated kinase (ERK) cascade (120, 121). Of note, hypoxia is also known to affect macrophage polarization and especially hypoxia-inducible factors were extensively studied as important regulators in this process (122, 123). Since hypoxia also alters type I IFN production and signaling in the TME, type I IFNs might act as a potential mediator linking hypoxia and macrophage polarization.

In conclusion, the effect of type I IFNs on TAM polarization appears to be highly context-dependent, however, a skewing towards a pro-inflammatory, classically activated, M1-like phenotype, associated with anti-tumor properties, was more frequently observed. Nevertheless, the application of type I IFNs as therapeutic measure to re-build a sufficient anti-tumor immunity has to be evaluated with caution.

### 3.3 Macrophage functions

In addition to general differentiation and polarization patterns, macrophages carry out highly specific functions in the tumor context, which are also affected by type I IFNs within the TME. In the following paragraphs, we will therefore provide a historical overview that shaped our current understanding of the impact of type I IFNs on killing and phagocytosis of tumor cells by macrophages (3.3.1). Furthermore, we will discuss type I IFN-dependent changes in the interplay between macrophages and other immune cells within the TME (3.3.2).

#### 3.3.1 Killing of tumor cells and phagocytosis

Macrophages are professional phagocytes and, thus, have the ability to take up particles, pathogens, but also cells (124). While phagocytosis of pathogens elicits pro-inflammatory responses [comprehensively reviewed in (125)], the uptake of apoptotic cells, also termed efferocytosis, triggers an anti-inflammatory or inflammation resolution phenotype in macrophages [for recent reviews see (126, 127)]. This mechanism is also exploited by tumors to polarize macrophages in the TME towards an anti-inflammatory and, thus, pro-tumorigenic phenotype after the uptake of apoptotic tumor cells (128). In theory, macrophages are also able to engulf non-apoptotic tumor cells given a sufficient receptor interaction. Respective phagocytic receptors on the surface of macrophages are for example MerTK, mannose receptors, triggering receptor expressed on myeloid cells 2 (TREM2), and CD36 (125). During early malignant transformation, tumor cells commonly express “eat-me” signals like calreticulin and phosphatidylserine, or present altered antigen profiles, which allows for recognition and clearance by phagocytes (129). However, during the process of immune evasion tumor cells increasingly display “don’t-eat-me” signals, i.e., phagocytosis checkpoints, like programmed cell death 1 ligand 1 (PD-L1, CD274), MHC-I, and CD47, to tune down anti-tumor immune responses (130, 131). Not surprisingly, therapeutic efforts targeting the CD47/signal-regulatory protein α (SIRPα) and the PD-L1/PD-1 axes for cancer immunotherapy show promising results (132). It is questionable though, whether macrophages alone are able to eradicate a whole tumor, since tumor regression caused by approaches to enhance macrophage phagocytosis (e.g., using anti-CD47) often requires intact T cell responses (129). Nevertheless, it was already shown more than 50 years ago that macrophages indeed are able to kill tumor cells both *in vitro*, for example after immunization against the target cells (133), as well as *in vivo*, e.g., in combination with opsonizing antibodies or upon stimulation with inflammatory mediators (134).

During the 1970s, first reports demonstrated that IFNs affect anti-tumor properties of macrophages by increasing their phagocytosis activity, but also by enhancing their ability to kill or inhibit growth of tumor cells (135–137), which was confirmed by numerous studies using combinations of monocytes and macrophages with different tumor cell lines *in vitro* (138–141). Notably, in some of those early reports the exact IFN species was not determined or the experimenters made use of purified human fibroblast IFN, which was claimed to be IFN-β. Later, Webb and colleagues demonstrated that various IFN-α species had similar, but not identical capacities to enhance macrophage cytotoxicity (142). Already early on, it was shown that the tumoricidal capacity of peripheral blood monocytes from tumor patients was similarly increased by type I IFNs *in vitro* (143, 144). In parallel, type I IFNs were demonstrated to enhance phagocytosis of opsonized and non-opsonized bacteria or erythrocytes by peritoneal macrophages, indicating a general enhancement of the phagocytic capacity of macrophages by type I IFN (145, 146). Yet, in contrast to the *in vitro* findings, phagocytosis and tumor cell killing appeared to be unaffected or reduced in macrophages from patients that were treated with type I IFNs. For instance, phagocytosis of yeast particles was not altered in peripheral monocytes from cancer patients after short-term IFN-α treatment, and even decreased after longer treatment regimens (147). Similarly, cytotoxicity of monocytes from cancer patients treated with type I IFN towards tumor cells was not altered (148, 149). The marked differences between *in vitro* and *in vivo* effects of type I IFNs were further corroborated by a study showing that monocytes from patients with untreated renal carcinoma exerted increased monocyte antibody-dependent cellular cytotoxicity (ADCC) after *in vitro* IFN-β treatment, while monocytes isolated 4 hours after IFN-β treatment of patients displayed decreased ADCC activity (150).

It became increasingly clear, that the effect of type I IFNs on the tumor killing capacities of macrophages was not a single-player game, but resulted from a complex network of interactions between different pathogens, alarmins, and cytokines. Pace and co-workers were the first to question the solitary action of type I IFNs in boosting macrophage cytolytic activity, as they observed the requirement of a second stimulus to induce tumor-cell killing by type I IFN-primed mouse peritoneal macrophages (151). Moreover, a cytotoxicity-promoting effect of type I IFNs was observed when combined with one or multiple other stimuli (152). Surprisingly, IFN-β appeared to attenuate the cytotoxicity-promoting effect of other mediators, like LPS or IFN-γ, in macrophages (153, 154). Nevertheless, there was accumulating evidence that type I IFNs are intrinsically involved in and critical for the tumoricidal activity of monocytes induced by numerous mediators, including M-CSF, LPS, and poly(I:C), but also for the spontaneous (untreated) tumoricidal activity of monocytes and macrophages (155–158).

Upon acceptance of the general concept that type I IFNs rather enhance tumor cell killing by macrophages, nitric oxide (NO) emerged as critical mediator contributing to the anti-tumor properties of the type I IFNs. In fact, while IFN-β was found to strongly enhance LPS-mediated iNOS expression and tumoricidal NO production in macrophages, LPS-mediated NO production by itself appeared to depend on type I IFNs (159–163). Pre-treatment with type I IFN also increased NO production and cytotoxicity in peritoneal macrophages upon contact with glioma cells (164). In recent years, the stimulating effect of type I IFNs on the tumoricidal activity of macrophages was consolidated. For example, Shime et al. observed that F4/80^+^ macrophages isolated from LLC displayed elevated cytotoxicity against LLC cells after activation of TLR3 signaling by poly(I:C) injection (165). Moreover, overexpression of IFN-β in iPSC-derived macrophages enhanced their cytotoxic activity against metastatic melanoma cells (101). In line, pre-treatment of BMDMs with IFN-α and -β further enhanced poly(I:C)-induced iNOS expression and NO production, consequently limiting LLC growth (166).

In addition to the effects of type I IFNs on the cytotoxic capacity of monocytes and macrophages, their impact on the phagocytic activity is also controversially discussed. On the one hand, the uptake of antigen or unopsonized fungal particles was reduced in monocyte-derived macrophages after exposure to IFN-β (167). In line, treatment of BMDMs with IFN-α decreased the engulfment of pHrodo particles (168). On the other hand, treatment of murine BMDMs with a STING inhibitor markedly reduced the amount of engulfed pHrodo bioparticles (169). Of note, in this context activation of STING resulted from the uptake of apoptotic cells. Along these lines, in a murine zymosan-induced peritonitis model peritoneal macrophages from IFN-β KO mice displayed reduced efferocytosis of apoptotic cells compared to macrophages from WT cells (170) indicating that IFN-β is involved in efferocytosis. An enhanced phagocytic capacity was also observed in CD169^+^ monocyte-derived macrophages upon IFN-α or -β stimulation. Specifically, CD169^+^ monocytes engulfed more apoptotic hepatoma cells than monocytes with low CD169 expression (105). Strikingly, type I IFN-mediated changes in macrophage phagocytic activity not only occur in monocyte-derived macrophages, but also in resident macrophage populations. For instance, Escoubas and co-workers identified a type I IFN-responsive microglia population characterized by elevated expression of ISGs, which engulfed more neurons compared to other microglia populations. Moreover, upon injection of IFN-β into the mouse brains, the number of phagocytic microglia increased, while the opposite was observed in brains of IFNAR-deficient mice (171).

In conclusion, type I IFNs enhance both tumoricidal and phagocytic capacities of monocytes and macrophages in the TME, and thus, could open new avenues for macrophage-based cancer therapies.

#### 3.3.2 Shaping the tumor immune milieu

Apart from their direct impact on tumor cells, macrophages execute important functions as immune modulators, thereby coordinating the response of other immune cells. In addition to presenting tumor antigens to activate T cell responses, macrophages communicate with their environment largely via the release of cytokines, i.e., secreted proteins responsible for the recruitment, proliferation, differentiation, and polarization of other cells. The set of cytokines released is highly dependent on the specific macrophage phenotype. Classically activated, pro-inflammatory macrophages rather secrete inflammatory cytokines, such as TNF-α, IL-1β, IL-6, and IL-12, which are involved in the induction of acute inflammatory responses with vascular permeabilization, immune cell recruitment, fever, and acute phase protein production. On the contrary, the main anti-inflammatory cytokines released by alternatively activated macrophages, IL-10 and transforming growth factor (TGF)-β, antagonize the measures of the classical pro-inflammatory cytokines, e.g., they inhibit antigen presentation and suppress T cells or macrophages themselves. For more details, we refer readers to a comprehensive review (172).

Despite the fact that type I IFNs have been investigated for more than half a century, their effect on pro- and anti-inflammatory cytokine signatures is still not entirely clear, in part owing to a contradictory impact on the expression of certain cytokines, e.g., type I IFNs are generally accepted to upregulate both IL-12 and IL-10 [for an overview see (173)]. Already 40 years ago, it was observed that the pre-treatment of human monocytes with IFN-α or -β enhanced IL-1 secretion in response to endotoxins, while they did not induce IL-1 release by themselves (174). Type I IFNs further differentially affected expression and release of pro-inflammatory cytokines (IL-1α/β, IL-6, IL-12, TNF-α) in primary human and mouse macrophages dependent on the respective main stimulus. For example, while in CD14^+^ monocyte-derived macrophages pro-inflammatory cytokine expression in response to TLR3 [poly(I:C)]-, TLR2 (Pam3CSK)-, or TLR4 (Lipid A)-stimulation was attenuated by pre-conditioning with IFN-β, the degree of inhibition varied massively between the stimuli, as it did for different response cytokines (175). Furthermore, the blockade of IFNAR increased pro-inflammatory cytokine expression induced by TLR4-activation, but not in the case of TLR7-agonism (176). Nevertheless, others also demonstrated a decrease in pro-inflammatory cytokine expression (IL-1β, IL-12, IL-18, TNF-α) in response to singular stimulation with IFN-α or -β, while the opposite was seen upon IFNAR depletion in primary human or mouse macrophages, or in differentiated THP-1 monocytes (170, 177, 178). In sum, type I IFNs, in contrast to type II IFNs, appear to mostly inhibit production of typical pro-inflammatory cytokines induced by various TLRs.

Interestingly, in the TME, stimulation of TAMs with type I IFNs appears to have the opposite effect. Of note, it is virtually impossible to determine the exact source of secreted cytokines, i.e., the specific cells releasing certain cytokines, within a complex interactive network such as the TME. So far, assignment of changes in cytokine production to certain cell types was often based on mRNA or intracellular protein expression, or data were generated based on semi-authentic TME models. As indicated above, Miyashita and colleagues found that iPSC-derived macrophages, which overexpress type I IFNs, display less TAM characteristics. In addition, they demonstrated increased secretion of pro-inflammatory cytokines from these cells (101). Likewise, *in vitro* treatment of whole PBMCs isolated from head and neck squamous cell carcinoma (HNSCC) patients with IFN-α2b increased the production of IL-12 in CD14^+^ monocytes and at the same time reduced expression of anti-inflammatory IL-4 and IL-10 (179). Along the same lines, activation of STING by mtDAMPs or cGAMP-liposomal nanoparticles increased mRNA expression of pro-inflammatory cytokines like TNF-α in BMDMs *in vitro*, but also in intratumoral CD11b^+^ cells *in vivo* (114, 180). Increased intratumoral TNF-α levels were also observed after poly(I:C) injection into 3LL lung tumors and macrophages were identified as source cells by intracellular protein staining. Gene expression analyses of F4/80^+^ cells isolated from tumors further revealed a general upregulation of pro-inflammatory cytokines, but also of IL-10 (165). In contrast, monocytes isolated from patients with primary breast cancer displayed a decreased production of TNF-α after stimulation with IFN-α (181).

Chemokines are a subgroup of cytokines which induce chemotaxis. The release of chemokines and the migration of cells towards the source of the signal is often induced by classical pro-inflammatory cytokines, but also by type I IFNs [for a comprehensive overview see (172)]. Furudate and co-workers observed marked changes in the production of numerous chemokines in IL-4-stimulated, monocyte-derived macrophages in response to IFN-α2a. Specifically, while CCL7, CCL8, CXCL10, CXCL11, and CXCL12 expression increased upon IFN-α2a treatment, expression of other chemokines, including CCL17, CCL18, CCL26, and CXCL2 was attenuated. While CXCL10 is originally described as an IFN-γ-induced chemokine, both CXCL10 and CXCL11 responded stronger to IFN-α2a stimulation (182). In line, we previously observed a marked increase in CXCL10 and CCL2 expression in monocytic cells upon hypoxia-induced, TLR4-mediated type I IFN signaling (73). These findings were corroborated by others, describing increased CXCL10 and CXCL11, and reduced CCL17, CCL18, and CCL20 expression in PBMC-derived macrophages upon IFN-β treatment (115). Of note, in contrast to our findings reduced CCL2 expression and secretion were observed in CD11b^+^ cells isolated from B16F10 melanomas of IFN-β-treated mice in this study. In TAMs isolated from 3LL tumors, CXCL10 was further upregulated in response to poly(I:C)-dependent type I IFN signaling (165). Likewise, increased mRNA expression of CCL5, CXCL10, and CXCL2 in TAMs of multiple myeloma-bearing mice was prevented by administration of a STING antagonist (180).

Importantly, differential chemokine expression patterns in the TME determine the exact composition of tumor immune cell infiltrates, and type I IFNs commonly contribute to increased secretion of chemokines from TAMs, especially of those recruiting T cells. Therefore, the question arises, if changes in macrophage type I IFN signaling influence recruitment or function of T cells. In fact, it has been known for a while that co-culture of T cells with peritoneal macrophages suppresses their proliferation and that this suppressive effect is overcome by the pre-treatment of macrophages with IFN-β (183, 184). Gianessi at al. recently demonstrated that the treatment of monocyte-derived macrophages with different IFNs affected size and marker expression of secreted small EVs and that these EVs were able to alter T cell phenotype and cytokine expression (185). Moreover, in a type 1 diabetes mouse model macrophage-specific deletion of IFNAR attenuated autoreactive T cell responses, especially the infiltration of autoreactive T cells into islets, and thereby progression of the disease (186). There is also evidence that type I IFN signaling in macrophages might play a role in the recruitment of T cells into the TME and contributes to intratumoral T cell activation and proliferation. Accordingly, myeloid-specific deletion of USP18, a negative regulator of type I IFN signaling, resulted in both increased infiltration of CD8^+^ T cells, especially central memory cells, into B16F10 melanomas and enhanced expression of activation markers (CD69, IFN-γ, perforin) in these T cells (85). Similarly, the increase of proliferative effector CD8^+^ granzyme B^+^ T cells in the pancreatic ductal adenocarcinoma (PDAC) TME after treatment with a CD11b agonist was attributed to the induction of a STING-dependent type I IFN signature in TAMs (187). Moreover, the increase in CD8^+^ T cell numbers in head and neck tumors after TGF-β inhibition was attenuated upon blockade of IFNAR, suggesting that macrophage-derived type I IFN augmented T cell-mediated anti-tumor immunity. This notion was further supported by the observation that treatment of irradiated RAW264.7 macrophages or BMDMs with TGF-β strongly reduced IFN-β release, while TGF-β receptor 2 (TGFβR2) neutralization increased it (188). In a similar direction, Affandi and colleagues reported enhanced antigen presentation of IFN-α-pre-treated monocytes to antigen-specific CD8^+^ T cells (106). Notably, another study demonstrated that the tumor-specific polarization of monocytes towards an immunostimulatory phenotype, was associated with increased expression of a type I IFN signature. Specifically, monocytes polarized by type I IFN-secreting tumor cells expressed ISGs and had a higher capacity to stimulate T cell proliferation, which again was prevented by IFNAR blockade. At the same time, an immunosuppressive monocyte sub-population suppressed T cell proliferation, that was overcome by IFN-β treatment (189). Similarly, CD14^+^ monocytes that were treated with supernatants of HCC cells and IFN-α2 increased T cell proliferation and the production of T cell-derived IFN-γ upon co-culture (105). Nevertheless, while there is compelling evidence for enhanced T cell recruitment in the TME due to type I IFN responses in macrophages, opposite results have also been reported: co-culture of splenic T cells with PDAC tumor cells and IFN-α-treated immortalized BMDMs (iBMDMs) *in vitro* increased T cell apoptosis compared to co-culture with untreated iBMDMs. Moreover, iBMDMs treated with IFN-α prior to orthotopic co-injection with PDAC tumor cells markedly decreased the infiltration of CD4^+^/CD8^+^ T cells and increased the percentage of exhausted T cells in the TME (168).

In addition to the recruitment of T cells, macrophages also control T cell activity, amongst others via the PD-1/PD-L1 axis. Specifically, PD-1 on T cells serves as receptor to suppress T cell activity upon interaction with its ligand PD-L1, which is expressed on professional antigen-presenting cells but also on tumor cells (190, 191). Nowadays, the expression of PD-L1 in the TME by tumor cells and immune cells is widely accepted and lead to the development of immune checkpoint inhibitor therapies (192–194). While IFN-γ is a potent inducer of PD-L1 expression, type I IFNs are also able to induce the expression of PD-L1 on tumor cells and immune cells like DCs or myeloid-derived suppressor cells (195–197). Similarly, there is also ample evidence for type I IFN-dependent expression of PD-L1 on monocytes and macrophages (105, 168, 198–200), and the type I IFN receptor IFNAR was found to be involved in both basal and stimulated PD-L1 expression in monocytes and macrophages (98, 201, 202). There are also sporadic reports suggesting that type I IFN signaling in macrophages might reduce PD-L1 expression instead. For example, a decreased proportion of PD-L1 expressing cells among isolated CD11b^+^ cells from B16F19 melanoma after IFN-β treatment was reported (115). Similarly, Lim and co-workers suggested decreased CD8^+^ T cell function upon deletion of IFNAR due to increased interaction of myeloid cells and T cells via the PD-1/PD-L1 axis in glioblastoma (99).

Importantly, besides their impact on T cells, macrophages influence the entire immune cell repertoire in the TME. Along those lines, myeloid-specific KO of Gasdermin D (GSDMD) boosted anti-tumor immunity in anti-PD-L1-treated B16F10 melanoma-bearing mice, as characterized by elevated infiltration of CD4^+^ and CD8^+^ T cells, but also B cells and natural killer (NK) cells. Furthermore, increased expression of the pro-inflammatory mediators TNF-α and IFN-γ and decreased expression of immune checkpoints were observed. Based on scRNA-seq data suggesting a strong type I IFN response in GSDMD-deficient macrophages, in combination with the finding that global cGAS deletion prevented the aforementioned effects, the authors proposed cGAS-dependent type I IFN response in macrophages to be responsible for the observed anti-tumor immune response (203). Similarly, myeloid-specific deletion of STING reduced number, proportion, and perforin expression of NK cells in the MC38 liver metastasis model. Moreover, enhanced expression of NK cell co-stimulatory ligands on BMDMs in response to co-culture with MC38 tumor cells, was reduced in STING KO macrophages (204). In contrast, Platsoucas and colleagues reported early on that monocytes were not involved in IFN-induced cytotoxicity of NK cells (205).

Taken together, despite contradictory findings, most current reports indicate that macrophages with established type I IFN responses shape the TME such that it promotes anti-tumor immunity. In particular, type I IFN-stimulated macrophages attract and activate T cells to establish a tumor-suppressive environment.

## 4 Tumor therapeutic perspectives

As discussed above, type I IFNs elicit mostly anti-tumorigenic effects on monocytes and macrophages within the TME. Specifically, type I IFN responses within myeloid cells inhibit monocyte-to-TAM differentiation, promote pro-inflammatory polarization, enhance tumor cell killing, and positively affect anti-tumor functions of other immune cells, most importantly T cells, during their interaction with macrophages (**Figure 3**). While several tumor-intrinsic features enhance the production of type I IFNs, the resulting selection pressure forces tumors to evolve adaptive mechanisms to attenuate or prevent type I IFN production (**Figure 2**).

Considering that elevated type I IFN concentrations in the TME often correlate with favorable prognosis and outcome, direct administration of type I IFNs, but also the stimulation of endogenous type I IFN production, or selective induction of specific ISG subsets emerged as promising tumor therapeutic concepts (206). Nevertheless, administration of exogenous type I IFNs as alternative or adjuvant tumor therapy was previously tested primarily based on the effects of type I IFNs on other immune cells as well as on their cytostatic impact on tumor cells [for a comprehensive overview of the use of IFNs in oncology in the last century, see (207)]. While IFN-α2a and IFN-α2b are already approved for clinical use, there are numerous clinical trials ongoing, testing if IFN-α, when combined with chemotherapy or immune checkpoint blocker therapy, might have beneficial effects (208). Importantly though, systemic administration of recombinant type I IFNs is often overshadowed by excessive toxicity and auto-immune reactions (209). Similarly, STING agonists are currently tested for their therapeutic potential in early clinical studies, though the exact toxicity profiles remain to be determined (210). Thus, it appears compelling to selectively target IFN responses in macrophages within the TME to unleash or reinstate anti-tumor macrophage functions, at the same time preventing unwanted adverse effects of systemic treatments. Apart from the direct administration of recombinant type I IFNs, various therapy approaches have been shown to elicit type I IFN responses. Most prominently, therapies triggering an immunogenic cell death (ICD) response, i.e., a programmed cell death, which activates the immune system, are characterized by elevated type I IFN production. In fact, the release of type I IFNs even serves as a biomarker for successful induction of ICD (211). Mechanistically, ICD-inducers, such as selected chemotherapies, radiotherapy, and targeted anticancer agents, foster the release of intra- or extracellular DAMPs, which in turn promote type I IFN responses specifically in innate immune cells with high PRR expression, such as monocytes and macrophages (212, 213). In line, after radiotherapy, tumor cells and hematopoietic cells produce type I IFNs, probably due to sensing of dsDNA derived from irradiated tumor cells (214, 215) or due to micronuclei, which emerge upon mitotic progression after radiotherapy-induced dsDNA breaks (216). Similarly, therapy with Tumor Treating Fields (TTFields), an FDA-approved therapy for glioblastoma and malignant mesothelioma, which applies low-energy alternating electric fields causing interruption of mitosis and formation of micronuclei, was shown to induce type I IFNs and ISGs (217). In either case, type I IFNs appeared to contribute to anti-tumor immune responses. While the intrinsic mechanisms described in the first part of this review induce moderate levels of type I IFN only, it can be assumed that type I IFNs increase to much higher levels in response to cell death-inducing therapies due to the massively increased amounts of released self-nucleic acids and other DAMPs. Of note, while a physiological type I IFN induction is considered to be anti-tumorigenic, chronically elevated type I IFN levels in tumors commonly lead to high expression of a subset of ISGs termed “IFN-related DNA damage resistance signature” (IRDS), which contributes to the development of therapy resistances (218). Rapid and strong induction of type I IFNs by therapeutic interventions again fosters the anti-tumorigenic properties [for a comprehensive overview over the role of type I IFN levels in the balance between pro- and antitumor effects we refer interested readers to (7)]. The IRDS signature resembles the antiviral response mediated via enhanced expression of STAT1/2 and IRF9 proteins, which even in an unphosphorylated state allow for expression of some ISGs (219). While IRDS induction in tumors appears to be partly understood, it can be speculated that analogous resistance mechanisms in response to chronic exposure to low levels of type I IFNs might exist in macrophages as well. Here, they could counter pro-inflammatory polarization and anti-tumor functions induced by acute type I IFN stimulation. Thus, reactivation of pronounced type I IFN responses in myeloid cells by overcoming tumor immune evasion mechanisms, e.g., via degradation of nucleic acids or downregulation of sensing pathways, could add to the therapeutic exploitation of the anti-tumor functions of type I IFN-stimulated macrophages. Along these lines, tumor microenvironmental factors, such as hypoxia or metabolic intermediates, affect type I IFN responses in a highly cell type-specific manner. This specificity might be used to selectively enhance IFN-dependent anti-tumor activities of macrophages.

Taken together, since macrophages play a decisive role in tumor development and are amongst the primary responders to IFNs, selectively altering type I IFN responses in macrophage to reinstate anti-tumor functions emerges as an attractive concept for future tumor therapeutic endeavors.
